# Supplementary material for: Content-rich biological network constructed by mining PubMed abstracts
Source: BMC Bioinformatics. 2004 Oct 8;5:147. doi: 10.1186/1471-2105-5-147 (PMC528731; doi:10.1186/1471-2105-5-147)
Supplement: Additional File 2 — The original results of the above study (non-essential files are deleted to keep the file size under the limit set by BMC bioinformatics). [file 1471-2105-5-147-S2.bz2 › chilibotAdditionalFile2/dip50/722ID2167176E18062/html/SECE_SECY.html]

 


 **SECE** and **SECY** 
  
Found 94 abstracts in PubMed, retrieved 50.  
 

 What does Google say? 
 PDF only 
| .edu only 

---

**Interactive relationship** (e.g. stimulation, inhibition, etc)

**Neutral relationship**- Although the importance of interactions involving both the cytosolic and transmembrane regions of  **SecY**  and  **SecE**  has been documented, no information has been available for the physical contact sites of these translocase subunits in their cytosolic domains.  Ref: 12809500 Biochemistry, 2003
- Thus, Syd acts against the SecY24 form of translocase, in which  **SecY**   **SecE**  interaction has been compromised, to exclude the SecA motor protein from the SecYE channel complex.  Ref: 9668058 J Biol Chem, 1998
- Previously, this mutation was shown to impair the interaction between  **SecY**  and  **SecE** , the two fundamental subunits of the membrane embedded part of protein translocase.  Ref: 9668058 J Biol Chem, 1998
- Mapping the sites of interaction between  **SecY**  and  **SecE**  by cysteine scanning mutagenesis.  Ref: 11445571 J Biol Chem, 2001
- Both genetic and biochemical data indicate interactions of several transmembrane segments TMSs of  **SecY**  with  **SecE** .  Ref: 11445571 J Biol Chem, 2001
- Unique cysteines were introduced into transmembrane segment TMS 2 of  **SecY**  and TMS 3 of  **SecE**  to probe possible sites of interaction between the integral membrane subunits.  Ref: 10413486 Biochemistry, 1999
- We have introduced cysteine residues within the first periplasmic loop of  **SecY**  and the second periplasmic loop of  **SecE** , at a specific pair of positions identified by this genetic interaction.  Ref: 10348856 J Bacteriol, 1999
- By exploiting the salt insensitive interaction of the cellulose binding domain CBD of the Clostridium thermocellum cellulosome with cellulose, purification of CBD fused versions of  **SecY**  and  **SecE** , components of the translocation apparatus of the halophilic archaeon Haloferax volcanii, was undertaken.  Ref: 12579382 Extremophiles, 2003
- We suggest that the cytosolic and intramembrane interactions bring these two subunits together, forming a functionally crucial SecYE interface involving the  **SecY**  C5 region and the conserved cytosolic segment of  **SecE** .  Ref: 12809500 Biochemistry, 2003
- Thus, CBD cellulose interactions can be employed as a salt insensitive affinity purification system for the capture of complexes containing the Haloferax volcanii translocation apparatus components  **SecY**  and  **SecE** .  Ref: 12579382 Extremophiles, 2003
- Deletion of the N terminal region of  **SecE**  also rendered the  **SecE**   **SecY**  SecG complex unstable.  Ref: 11129584 Biosci Biotechnol Biochem, 2000
- Chloroplast  **SecY**  is complexed to  **SecE**  and involved in the translocation of the 33 kDa but not the 23 kDa subunit of the oxygen evolving complex.  Ref: 10207046 J Biol Chem, 1999
- His6  **SecY**  brought down both  **SecE**  and SecG, as expected.  Ref: 9180258 FEBS Lett, 1997
- However, anti HA immunoprecipitates contain neither untagged  **SecE**  nor  **SecY**  cross linked to  **SecE** .  Ref: 10944122 EMBO J, 2000
- ii Cross linking of membranes containing unmodified  **SecE**  and hemagglutinin tagged  **SecE**   **SecE**  HA reveals cross links between  **SecY**  and  **SecE**  and between  **SecY**  and  **SecE**  HA.  Ref: 10944122 EMBO J, 2000
- Cysteine directed cross linking demonstrates that helix 3 of  **SecE**  is close to helix 2 of  **SecY**  and helix 3 of a neighboring  **SecE** .  Ref: 10413486 Biochemistry, 1999
- However, the SecG dependent stabilization of  **SecY**  was less potent than the  **SecE**  dependent stabilization.  Ref: 8526914 Biochem Biophys Res Commun, 1995
- Remarkably, this model reveals that TMS 3 of  **SecE**  is strongly tilted relative to  **SecY** .  Ref: 12138117 J Biol Chem, 2002
- These results indicate that SecG preferentially interacts with  **SecE** , which associates with  **SecY** , the SecG  **SecE**   **SecY**  complex thus being formed in the cytoplasmic membrane.  Ref: 8526914 Biochem Biophys Res Commun, 1995
- The stabilization of  **SecY**  overproduction by Va  **SecE**  was as effective as that by Ec  **SecE** , indicating that Va  **SecE**  interacts with E. coli  **SecY**  despite the difference in energy requirement.  Ref: 9791006 Biochem Biophys Res Commun, 1998
- These results indicate that residue 240 of  **SecY**  is crucial for the interaction between the cytosolic domains of  **SecY**  and  **SecE**  required for the establishment of the translocase complex.  Ref: 9645430 Mol Gen Genet, 1998
- The E. coli enzyme is required for proteolytic degradation of some unstable proteins that include both soluble regulatory proteins such as sigma 32 heat shock sigma factor and phage lambda CII transcriptional activator, and membrane proteins including uncomplexed forms of  **SecY**  forms the translocon together with  **SecE**  and SecG and the a subunit of the F0 complex of the H ATPase.  Ref: 10077851 FEMS Microbiol Rev, 1999
- Furthermore, the secY241 mutation seemed to represent a hyper reversion with respect to the  **SecY**   **SecE**  interaction.  Ref: 9645430 Mol Gen Genet, 1998
- In contrast, overproduction of Syd is toxic to the secY24 mutant, which shows a partial defect in  **SecY**   **SecE**  interaction.  Ref: 9645430 Mol Gen Genet, 1998
- Although His6  **SecE** , expressed in wild type cells, brought down both  **SecY**  and SecG, neither of them was brought down when the same protein was expressed in the secY24 mutant cells.  Ref: 9180258 FEBS Lett, 1997
- A chimeric  **SecE**  possessing the cytoplasmic region of the E. coli  **SecE**  and the following region of the Bacillus subtilis  **SecE**  was able to form the translocation machinery together with SecA,  **SecY** , and SecG.  Ref: 8827448 J Biochem Tokyo , 1996
- These mutants, as well as previously constructed mutations in the third membrane spanning segment of  **SecE**  MSS3, were tested for their ability to complement a  **secE**  null mutation, for their effects on protein export in vivo, and for their ability to form a stable complex with  **SecY** .  Ref: 8702704 J Biol Chem, 1996
- In this report we show that cpSecE in cells depleted for bacterial  **SecE**  i supports growth, ii stabilizes, just like bacterial  **SecE** , the Sec translocase core component  **SecY** , and iii supports Sec dependent protein translocation.  Ref: 11389897 FEBS Lett, 2001
- In vivo analyses of interactions between  **SecE**  and  **SecY** , core components of the Escherichia coli protein translocation machinery.  Ref: 8702704 J Biol Chem, 1996
- These cross linkages were abolished by the Gly240 mutation in the  **SecY**  C4 region as well as by prlG alterations in  **SecE**  transmembrane segment 3, known to compromise  **SecY**   **SecE**  interaction.  Ref: 12809500 Biochemistry, 2003
- A  **SecE**  mutation that modulates  **SecY**   **SecE**  translocase assembly, identified as a specific suppressor of  **SecY**  defects.  Ref: 12533470 J Bacteriol, 2003
- To facilitate large scale purification of the SecYEG heterotrimer,  **SecY**  was fused at its amino terminus with a hexahistidine tag and co overexpressed with  **SecE**  and SecG.  Ref: 9425040 Biochemistry, 1998

**Non-interactive relationship** (e.g. studied together, co-existance, homology, etc.)

- Protein export in Escherichia coli is mediated by translocase, a multisubunit membrane protein complex with SecA as the peripheral subunit and the  **SecY** ,  **SecE** , and SecG proteins as the integral membrane domain.  Ref: 10074070 J Bacteriol, 1999
- By means of cysteine scanning mutagenesis, we have identified intermolecular sites of contact between TMS7 of  **SecY**  and TMS3 of  **SecE** .  Ref: 11445571 J Biol Chem, 2001
- Cross linked SecA copurified with hexahistidine tagged  **SecY**  and not with  **SecE** .  Ref: 9294424 J Bacteriol, 1997
- i Formaldehyde cross linking of translocase reveals cross links between  **SecY** ,  **SecE**  and SecG, but not higher order oligomers.  Ref: 10944122 EMBO J, 2000
- These results suggest that several regions contribute to the formation of a stable  **SecE**   **SecY**  complex but the elimination of a single contact point does not necessarily affect the functionality of the complex.  Ref: 8702704 J Biol Chem, 1996
- E. coli SecA, but not B. subtilis SecA, supported efficient ATP dependent translocation of the E. coli precursor OmpA proOmpA into inner membrane vesicles containing the hybrid SecYEG complexes, if E. coli  **SecY**  and either E. coli  **SecE**  or E. coli SecG were present.  Ref: 10559168 J Bacteriol, 1999
- Escherichia coli preprotein translocase comprises a membrane embedded hexameric complex of  **SecY** ,  **SecE** , SecG, SecD, SecF and YajC SecYEGDFyajC and the peripheral ATPase SecA.  Ref: 9305629 EMBO J, 1997
- The transport of large preproteins across the Escherichia coli plasma membrane is catalyzed by preprotein translocase, comprised of the peripherally bound SecA subunit and an integrally bound heterotrimeric domain consisting of the  **SecY** ,  **SecE** , and SecG subunits.  Ref: 7650029 J Biol Chem, 1995
- From the extent of SecG overproduction, the number of SecG molecules in one normal cell was estimated to be about 00, which is similar to those of  **SecY**  and  **SecE** .  Ref: 8526914 Biochem Biophys Res Commun, 1995
- **SecY**  and  **SecE**  are the two principal translocase subunits that create a channel like pathway for the transit of preprotein across the bacterial cytoplasmic membrane.  Ref: 12533543 J Biol Chem, 2003
- **SecY** , a subunit of protein translocase, is FtsH dependently degraded in vivo when it fails to associate with its partner the  **SecE**  protein.  Ref: 8940120 J Biol Chem, 1996
- Identification of a  **SecY**   **SecE**  Cytosolic Interface.  Ref: 12809500 Biochemistry, 2003
- These prokaryotic eukaryotic proteins include 1  **SecY**  Sec61alpha, 2  **SecE**  Sec61gamma, 3 SecG Sec61beta, 4 Ffh SRP54 and 5 FtsY SRP receptor subunit alpha.  Ref: 12507766 Biochim Biophys Acta, 2003
- We used hexahistidine tagged  **SecE**  and  **SecY**  to study how the core subunits  **SecY** ,  **SecE**  and SecG of Escherichia coli protein translocase interact with each other.  Ref: 9180258 FEBS Lett, 1997
- The majority are exported through pores formed by  **SecY**  and  **SecE** .  Ref: 11051763 Prog Nucleic Acid Res Mol Biol, 2001
- We now carried out site specific cross linking experiments to identify  **SecY**  and  **SecE**  regions that are physically close.  Ref: 12809500 Biochemistry, 2003
- SecYEG, a complex of integral membrane proteins  **SecY** ,  **SecE** , and SecG in the cytoplasmic membrane, constitutes a pathway for polypeptide movement.  Ref: 12812064 Yi Chuan Xue Bao, 2003
- **SecY** ,  **SecE**  and SecG form a membrane part of the protein translocation machinery.  Ref: 8526914 Biochem Biophys Res Commun, 1995
- Integral membrane components  **SecY** ,  **SecE** , and SecG of protein translocase form a complex in the Escherichia coli plasma membrane.  Ref: 12809499 Biochemistry, 2003
- These results confirm our previous conclusion that the secY24 alteration impairs the  **SecY**   **SecE**  interaction, and demonstrate that  **SecY**  and SecG can form a complex that does not contain  **SecE** .  Ref: 9180258 FEBS Lett, 1997
- Following transformation of Haloferax volcanii cells with CBD  **SecY**  or  **SecE**  encoding plasmids, cellulose based purification led to the capture of stably expressed, membrane bound 68 and 25 kDa proteins, respectively.  Ref: 12579382 Extremophiles, 2003
- Escherichia coli preprotein translocase contains a membrane embedded trimeric complex of  **SecY** ,  **SecE**  and SecG SecYEG and the peripheral SecA protein.  Ref: 9184221 EMBO J, 1997
- We have purified a translocationally active complex of the two subunits,  **SecY**  and  **SecE** , from Bacillus subtilis.  Ref: 9917412 J Mol Biol, 1999
- The cross linking of  **SecY**  to  **SecE**  demonstrates that these subunits are present in a one to one stoichiometry within the SecYEG complex.  Ref: 11445571 J Biol Chem, 2001
- The levels of  **SecY**  and SecG in the secEcsE501 mutant, which expresses  **SecE**  at a decreased level and is sensitive to low temperature, increased upon the expression of functional  **SecE**  derivatives, irrespective of the site of mutation, suggesting that the levels of  **SecY**  and SecG are co operatively determined by the level of functional, but not non functional,  **SecE** .  Ref: 8827448 J Biochem Tokyo , 1996
- On the basis of our model for  **SecY**  d1, these results suggest that the secY241 alteration enhances  **SecY**   **SecE**  interaction.  Ref: 9645430 Mol Gen Genet, 1998
- For both substrates, the mRNA levels of secA, secD,  **secE** , secF, and  **secY**  genes were almost constant during the early and log phases, but showed a marked decrease at the beginning of the stationary phase followed by a full recovery of mRNA level in the late stationary phase.  Ref: 10696484 Can J Microbiol< MedlineTA>Can J Microbiol, 1998
- To systematically determine the functional exchangeability of translocase subunits, all of the relevant combinations of the E. coli and B. subtilis  **secY** ,  **secE** , and secG genes were expressed in E. coli.  Ref: 10559168 J Bacteriol, 1999
- Preprotein translocation in Escherichia coli is mediated by the translocase with SecA as peripheral ATPase and  **SecY** ,  **SecE** , and SecG as membrane domain.  Ref: 9425040 Biochemistry, 1998
- By analogy with  **secY**  prlA, secA prlD, and  **secE**  prlG, secG could therefore be called secG prlH.  Ref: 9020118 J Biol Chem, 1997
- Precursor protein translocation across the Escherichia coli inner membrane is mediated by the translocase, which is composed of a heterotrimeric integral membrane protein complex with  **SecY** ,  **SecE** , and SecG as subunits and peripherally bound SecA.  Ref: 9294424 J Bacteriol, 1997
- Cysteines introduced into  **SecY**  residue 244 in the fourth cytosolic domain C4 as well as into residues 354 356 and 362 in the C5 domain could be cross linked with natural or engineered residues at positions 79 and 81 in the central part of the cytosolic loop of  **SecE** .  Ref: 12809500 Biochemistry, 2003
- YidC could not be integrated in the absence of  **SecY** ,  **SecE** , or SecG, suggesting that YidC, in contrast to its mitochondrial orthologue Oxa1p, cannot engage a SecYEG independent protein conducting channel.  Ref: 11777926 J Biol Chem, 2002
- Likewise,  **SecY**   **SecE**  complex could be isolated from the secG deleted strain.  Ref: 9180258 FEBS Lett, 1997
- Bacterial protein translocation is mediated by translocase, a multisubunit membrane protein complex that consists of a peripheral ATPase SecA and a preprotein conducting channel with  **SecY** ,  **SecE** , and SecG as subunits.  Ref: 10559168 J Bacteriol, 1999
- Although cross linking reversibly inactivates translocation, the contact between TMS7 of  **SecY**  and TMS3 of  **SecE**  remains unaltered upon insertion of the preprotein into the translocation channel.  Ref: 11445571 J Biol Chem, 2001
- The  **SecY**  and  **SecE**  single Cys mutants were cloned individually and in pairs into a secYEG expression vector and functionally overexpressed.  Ref: 10413486 Biochemistry, 1999
- We have now placed the  **secY** ,  **secE** , and secG genes under the control of an arabinose inducible promoter on a multicopy plasmid.  Ref: 7650029 J Biol Chem, 1995
- Co immunoprecipitations of  **SecE**  and  **SecY**  revealed that all mutant proteins, except those altered in PD2, destabilized the  **SecE**   **SecY**  complex.  Ref: 8702704 J Biol Chem, 1996
- We previously described a dominant negative  **secY**  d1 allele in Escherichia coli, whose product interferes with protein export, presumably by sequestering  **SecE** , the stabilizing partner of  **SecY** .  Ref: 9645430 Mol Gen Genet, 1998
- In the bacterial cytoplasmic membrane, the channel is formed by oligomers of a heterotrimeric membrane protein complex consisting of subunits  **SecY** ,  **SecE**  and SecG.  Ref: 12167867 Nature, 2002
- Mapping an interface of  **SecY**  PrlA and  **SecE**  PrlG by using synthetic phenotypes and in vivo cross linking.  Ref: 10348856 J Bacteriol, 1999
- **SecY**  and  **SecE**  are integral cytoplasmic membrane proteins that form an essential part of the protein translocation machinery in Escherichia coli.  Ref: 10348856 J Bacteriol, 1999
- In the gram positive bacterium Bacillus subtilis, SecA,  **SecY** , and  **SecE**  have been identified through genetic analysis.  Ref: 10074070 J Bacteriol, 1999
- the two essential membrane components  **SecY**  and  **SecE**  are sufficient for their formation.  Ref: 9917412 J Mol Biol, 1999
- We now demonstrate that  **SecE**  also contacts TMS 10 of  **SecY** .  Ref: 12138117 J Biol Chem, 2002
- The most intriguing observation is that translocation of the TMAO reductase across the cytoplasmic membrane is independent of the  **SecY** ,  **SecE** , SecA and SecB proteins.  Ref: 9427745 EMBO J, 1998
- Overproduction of SecG stabilized the overproduction of  **SecE**  C, an unstable truncated derivative of  **SecE** , as effectively as  **SecY**  does.  Ref: 8526914 Biochem Biophys Res Commun, 1995
- Since  **SecY**  could not be overproduced without  **SecE** , these data indicate a stable interaction between the heterologous  **SecY**  and  **SecE**  subunits.  Ref: 10559168 J Bacteriol, 1999
- Previous cysteine scanning studies showed that the transmembrane segment TMS 3 of  **SecE**  contacts TMS 2 and 7 of  **SecY** , and TMS 3 of another  **SecE** .  Ref: 12138117 J Biol Chem, 2002
- ... the  **SecY**   **SecE**  complex without SecG dissociated at a slightly lower temperature 20 23 degrees C.  Ref: 9180258 FEBS Lett, 1997
- Translocation occurs at a multisubunit membrane bound enzyme termed translocase, which consists of a peripheral preprotein binding site and an ATPase domain termed SecA, a core heterotrimeric integral membrane protein complex with  **SecY** ,  **SecE**  and SecG as subunits, and an accessory integral membrane protein complex containing SecD and SecF.  Ref: 10066476 Curr Opin Microbiol, 1998
- Subunit interactions in the Escherichia coli protein translocase  **SecE**  and SecG associate independently with  **SecY** .  Ref: 9180258 FEBS Lett, 1997
- In contrast, MelB membrane assembly does not require SecA,  **SecY**  or  **SecE** , all three proteins which are strictly required for protein translocation.  Ref: 8895565 EMBO J, 1996
- We now report that both prlA and prlG mutations weaken the associations between the  **SecY** ,  **SecE**  and SecG subunits of the translocase.  Ref: 10369667 EMBO J, 1999
- Overproduction of  **SecE** , but not of  **SecY** , alleviated the dominant negative effects.  Ref: 12655407 Mol Genet Genomics, 2003
- These data establish that  **SecY** ,  **SecE** , and SecG constitute the integral membrane domain of preprotein translocase.  Ref: 7650029 J Biol Chem, 1995
- Evidence indicates that the Ser105 to Pro alteration in the C terminal transmembrane segment of  **SecE**  weakens  **SecY**   **SecE**  association.  Ref: 12533470 J Bacteriol, 2003
- Oxidation of the single Cys pairs revealed periodic contacts between  **SecY**  and  **SecE**  that are confined to a specific alpha helical face of TMS 2 and 3, respectively.  Ref: 10413486 Biochemistry, 1999
- Whereas homologues of Sec61alpha  **SecY**  and Sec61gamma  **SecE**  exist in archaea, identification of the third component Sec61beta or SecG has remained elusive.  Ref: 11943537 Trends Biochem Sci, 2002
- **SecY** ,  **SecE**  and SecG form a heterotrimer, which functions as a protein translocation channel in Escherichia coli.  Ref: 12655407 Mol Genet Genomics, 2003
- In the present work, we demonstrate that a  **SecE**  homologue is localized to the thylakoid membrane, where it forms a complex with cpSecY  [ **SECY** ] .  Ref: 10207046 J Biol Chem, 1999
- An artificially constructed TM9 PhoA protein, derived from  **SecY** , as well as the  **SecE**  protein, were sensitized to FtsH by addition of extra amino acid sequences to their N terminal cytosolic tails.  Ref: 11256624 EMBO Rep, 2000
- Isolation of fusion proteins containing  **SecY**  and  **SecE** , components of the protein translocation complex from the halophilic archaeon Haloferax volcanii.  Ref: 12579382 Extremophiles, 2003
- A reducible  **SecY**   **SecE**  complex is also observed, demonstrating that these amino acids must be sufficiently proximal to form a disulfide bond.  Ref: 10348856 J Bacteriol, 1999
- The stepwise mode of polypeptide insertion was also observed with reconstituted proteoliposomes comprising purified SecA,  **SecY** , and  **SecE** .  Ref: 9038205 J Biol Chem, 1997
- In a fusion protein in which the intact  **SecY**  protein was fused to the alpha fragment, stabilization of the  **SecY**  part by overproduction of the partner  **SecE**  protein resulted in an increased alpha complementation activity of beta galactosidase.  Ref: 7487977 Biochem Biophys Res Commun, 1995
- **SecE** , along with  **SecY** , form a complex in the cytoplasmic membrane essential for protein translocation.  Ref: 8702704 J Biol Chem, 1996
- Escherichia coli preprotein translocase comprises a membrane embedded trimeric complex of  **SecY** ,  **SecE**  and SecG.  Ref: 11867527 EMBO J, 2002
- While  **SecE**  and  **SecY**  are generally accepted as essential components of translocase, the role of SecG is more ambiguous.  Ref: 10732681 Mol Gen Genet, 2000
- **SecY** ,  **SecE**  and SecG form the membrane embedded core complex of the Escherichia coli protein export apparatus.  Ref: 10732681 Mol Gen Genet, 2000
- Overproduction of the integral subunits of the translocase, the  **SecY** ,  **SecE**  and SecG polypeptides, results in an increased level of membrane bound SecA.  Ref: 8951810 Mol Microbiol, 1996
- The integral membrane proteins  **SecY**  and  **SecE**  are conserved core subunits of the translocase.  Ref: 12138117 J Biol Chem, 2002
- We conclude that each of  **SecE**  and SecG independently binds to  **SecY** , the central subunit of protein translocase, although the trimeric complex is more stable than the binary complexes.  Ref: 9180258 FEBS Lett, 1997
